# Maternal and fetal outcomes of pregnancy in chronic kidney disease: diagnostic challenges, surveillance and treatment throughout the spectrum of kidney disease

**DOI:** 10.1590/2175-8239-JBN-2020-0055

**Published:** 2021-01-11

**Authors:** Inês Filipe Gouveia, Joana Raquel Silva, Clara Santos, Claudina Carvalho

**Affiliations:** 1 Serviço de Ginecologia e Obstetrícia do Centro Hospitalar de Vila Nova de Gaia e Espinho, Portugal.; 2 Serviço de Nefrologia do Centro Hospitalar de Vila Nova de Gaia e Espinho, Portugal.

**Keywords:** Renal Insufficiency, Chronic, Pregnancy, Dialysis, Transplantation, Immunosuppressive Agents, Treatment Outcome., Insuficiência Renal Crônica, Gravidez, Diálise, Transplante, Imunossupressores, Resultado do Tratamento.

## Abstract

Pregnancy requires several physiological adaptations from the maternal organism, including modifications in the glomerular filtration rate and renal excretion of several products. Chronic kidney disease (CKD) can negatively affect these modifications and consequently is associated with several adverse maternal and fetal adverse outcomes (gestational hypertension, progression of renal disease, pre-eclampsia, fetal growth restriction, and preterm delivery). A multidisciplinary vigilance of these pregnancies is essential in order to avoid and/or control the harmful effects associated with this pathology. Dialysis and transplantation can decrease the risks of maternal and fetal complications, nonetheless, the rates of complications remain high comparing with a normal pregnancy. Several recent developments in this area have improved quality and efficacy of treatment of pregnant women with CKD. This article summarizes the most recent literature about CKD and pregnancy.

## Resumo

A gravidez requer várias adaptações fisiológicas do organismo materno, incluindo modificações na taxa de filtração glomerular e na excreção renal de vários produtos. A doença renal crônica (DRC) pode afetar negativamente essas modificações e, consequentemente, está associada a vários desfechos adversos maternos e fetais (hipertensão gestacional, progressão da doença renal, pré-eclâmpsia, restrição do crescimento fetal e parto prematuro). A vigilância multidisciplinar dessas gestações é fundamental para evitar e/ou controlar os efeitos deletérios associados a essa patologia. A diálise e o transplante podem diminuir os riscos de complicações maternas e fetais, no entanto, as taxas de complicações permanecem altas em comparação com uma gravidez normal. Vários desenvolvimentos recentes nesta área melhoraram a qualidade e a eficácia do tratamento de mulheres grávidas com DRC. Este artigo resume a literatura mais recente sobre DRC e gravidez.

## Introduction

The tendency for pregnancy at advanced maternal age and the increased diagnostic awareness of chronic kidney disease (CKD) during pregnancy have both contributed to the rising prevalence of gestations complicated by this disorder (estimated at 3% in high-income countries).^1^^,^ 2

The physiological modifications of renal function during pregnancy are critical for favorable pregnancy outcomes.^3^^-^ 5 Consequently, even in early stages of CKD, women are at risk of adverse maternal and fetal outcomes - pregnancy failure, pre-eclampsia (PE) / hemolysis, elevated liver enzymes and low platelets syndrome (HELLP), fetal growth restriction (FGR), preterm (PT) delivery, and progression to end-stage renal disease (ESRD). The risk for such complications increases along with the degree of renal dysfunction and further comorbidities such as diabetes, hypertension, and proteinuria.^6^^-^ 8

Due to the risks associated with CKD, a few older articles advocated this disease as a hazard to pregnancy independently of the severity of the disease, despite the current knowledge that milder stages have a better prognosis.^9^^,^ 10 The significant advances in perinatal and neonatal care changed this viewpoint drastically since the 1950s, and although there is increased risk for comorbidities even in mild stages, pregnancy is not contraindicated in the majority of patients with CKD.^8^^,^ 11

A multidisciplinary team that gathers the collaboration of obstetricians, nephrologists, dieticians, and neonatologists is essential for the surveillance and management of such pregnancies. Pre-conception planning plays a crucial role in the identification of the "window of opportunity" - the ideal period in which the woman's renal function is stabilized and further comorbidities are controlled.^6^^,^ 12

### Limitations of Current Evidence

Despite the increasing prevalence of CKD, there is lack of standardized criteria between studies and other aspects of this disease. Limitations of the current evidence are due to several factors:

1. Changes in the definition of CKD;
2. Insufficient number of studies of CKD in pregnancy and data regarding pre-pregnancy renal function;
3. Disease heterogeneity (affecting the pregnancy);
4. Inter-patient differences in progression of the disease;
5. Difficulties in the diagnosis of adverse outcomes such as PE due to the overlap of clinical features with CKD.

Despite the limitations, the objective of this review is to summarize the current recommendations regarding pregnancy in CKD and highlight recent advances and consensus regarding diagnosis and management of pregnancy adverse outcomes.^7^^,^ 13 ^,^ 14

## Discussion

### CKD in Pregnancy: Definition and Staging

CKD is broadly defined as any alteration in renal function, morphology, or imaging, or by a glomerular filtration rate (GFR) < 60 mL/min for a minimum of 3 months.^13^^-^ 15

With pregnancy, there is an increase of renal blood flow and physiological hyperfiltration, quantifiable as early as at 8 weeks of gestation. These adaptations lead to an increase in GFR and a decrease of serum creatinine level that can mask a decline in renal function.^5^^,^ 16

In non-pregnant individuals, the GFR can be estimated by different formulas - Cockcroft-Gault, Modification of Diet in Renal Disease (MDRD), and Chronic Kidney Disease Epidemiology Collaboration (CKD-EPI). Due to the physiological adaptation of pregnancy, both MDRD and CKD-EPI tend to underestimate GFR during this period. The Cockcroft-Gault formula can either under or overestimate this measure, especially in hypertensive pregnant women. Therefore, evaluation of renal function during pregnancy is limited to serial monitoring of serum creatinine. 1 ^,^ 13 ^,^ 17

Recent literature on this subject suggests that a serum creatinine superior to 0.87 mg/dL (77 mmol/L) should be considered outside the normal range for pregnancy, and these parameters can differ in different trimesters of pregnancy, indicating that trimester-specific limits should be applied.^17^ There is a trend to higher limits of creatinine in the first and third trimester compared to second trimester.^18^

### Maternal Impact of CKD

CKD can have different effects on the female reproductive system. CKD can disrupt the hypothalamic-pituitary-gonadal axis that controls the menstrual cycle and can also cause sexual dysfunction. 19

The decrease in glomerular filtration rate can influence sexual hormones, specifically the estrogen-mediated positive feedback, and the inhibition of the estradiol-stimulated LH surge seems to be the main factor responsible for anovulatory cycles and amenorrhea in women with CKD. 19 ^,^ 20

Sexual dysfunction etiology is complex, with both psychological and biological factors taking part. Some of the symptoms consist of decreased sexual activity and interest (frequently due to negative body image, even before dialysis), impairment of orgasm, insufficient vaginal lubrication, dyspareunia, and vaginismus.^19^^,^ 21 These effects can decrease the probability of pregnancy, although not unlikely.^22^^,^ 23

Infertility can also be a result of medication for treatment of underlying diseases of CKD, such as cyclophosphamide in lupus nephritis, an alkylating agent that leads to a decrease of developing follicles and, consequently, premature ovarian failure. The effect of this drug in fertility is age- and cumulative dose-dependent. 19 ^,^ 24

Fertility preservation techniques ought to be considered for women in childbearing age who wish to maintain the fertility for the future and are considered for treatment with cyclophosphamide. Options include oocyte, embryo, and ovarian tissue banking.^19^^,^ 24

Pregnancy in women with CKD, whether in early or late stages of the disease, has a higher risk of unfavorable outcomes (PE, FGR, PT delivery, fetal demise), even in the absence of proteinuria or hypertension. Pathologies such as PE and FGR are accountable for most cases of fetal demise, PT birth, and neonatal death in women with CKD.^20^^,^ 25 ^,^ 26 The probability for these complications increases with the progression of renal dysfunction and with the appearance of proteinuria and/or hypertension.^14^^,^ 27 ^,^ 28

Some of the drugs used in CKD women to slow the progression of renal disease, treat hypertension, or flares of specific underlying diseases (for example, lupus nephritis) can also be harmful for the fetus and some couse a demonstrated pattern of malformations.^29^^-^ 31 (Table 1)

**Table 1:** Immunosuppressants and other drugs commonly used in CKD patients during conception, pregnancy and lactation

DRUGS	Conception	Pregnancy			Lactation
	Safe/Unsafe	Safe/Unsafe	Maternal effects	Fetal effects	Safe/Unsafe
**IMMUNOSUPRESSIVE DRUGS**					
Corticosteroids	Safe	Safe (C)	Higher risk of bone loss diabetes, infection, preterm rupture of membranes	No teratogenicity. (No association to orofacial cleft in recent studies)	Safe (Monitor newborn)
Azathioprine	Safe	Safe (D)	Hyperkalemia, worsening hypertension and nephrotoxicity	No metabolization by the fetal liver	Safe
Cyclosporine	Safe	Safe (C)	Risk of hypertensive, hyperglycaemic and nephrotoxic effects	No teratogenicity. (reports of FGR and SGA)	Safe
Tacrolimus	Safe	Safe (C)	Similar to cyclosporine	No teratogenicity	Safe
Hydroxychloroquine	Safe	Safe (C)	Withdrawal may cause lupus flare.	No teratogenicity	Safe
**TO BE AVOIDED**					
m-Tor inhibitors (Sirolimus; Everolimus)	**Unsafe**	Unsafe (C)	Hyperlipidemia, hyperglycemia, nephrotoxicity	Unclear. (Toxic in animal studies)	Unclear
Mycophenolate	**Unsafe** (♀ and ♂ stop > 6 weeks before conception)	Unsafe (D)	Gastrointestinal symptoms and dose-related bone marrow suppression	Teratogenic. Severe fetal cardiovascular and cranial malformations	Unsafe (Excreted in breast milk)
Methotrexate	**Unsafe** (stop > 3 months before conception)	Unsafe (X)	Hepatotoxicity, gastrointestinal symptoms, alopecia, bone marrow suppression	Teratogenic	Unsafe (Excreted in breast milk)
Cyclophosphamide	**Unsafe** (♀ and ♂ stop > 3 months before conception)	Unsafe (D)	Affects ovarian function and fertility	Teratogenic. Congenital abnormalities of the skull, ear, face, limb and visceral organs, cytopenia	Unsafe (Excreted in breast milk. Discontinue breastfeeding during and for 36 hours after treatment)
**BIOLOGIC AGENTS**					
Rituximab	Unclear (Manufacturer advises to stop 1 year before conception)	Unclear	Active passage in the 2^nd^ and 3^rd^ trimester. Administer before or in early pregnancy.	Avoid unless potential benefits outweighs risks. (Potential risk of neonatal B cell depletion)	Unclear
Eculizumab	Unclear	Unclear	Monitor for increased dosage requirements	Active passage in the 2^nd^ and 3^rd^ trimester. No teratogenicity reported.	Unclear
Belimumab	Unclear (stop > 4 months before conception)	Unclear	Active passage in the 2^nd^ and 3^rd^ trimester. Administer before or in early pregnancy	Studies so far showed no teratogenicity. Limited data. Avoid unless potential benefits outweighs risks.	Unclear
OTHER DRUGS					
Aspirin	Safe	Safe (C)	Decreases risk of preeclampsia and FGR.	No teratogenicity	Safe
Low-molecular weight heparin	Safe	Safe (C)	Decreases risk of VTE	No teratogenicity	Safe
Allopurinol	Safe	Safe (C)	None	No teratogenicity	Excreted in breast milk. No reported adverse effects
Iron	Safe	Safe (B)	None	No teratogenicity	Safe
Erythropoietin	Safe	Safe (C)	Risk of hypertension.	No teratogenicity	Safe

Pre-conception optimization is highly important in order to lower the risk of adverse outcomes and have a successful pregnancy, accompanied by adequate surveillance.^13^^,^ 14 ^,^ 32

### Specific Renal Diseases

#### Primary Glomerulonephritis

The presence of isolated proteinuria or proteinuria associated with hematuria or hypertension may indicate the presence of renal disease.^32^^-^ 34 Due to the frequent urine analysis undertaken by women during pregnancy, glomerulonephritis can be diagnosed for the first time during this period. In women previously diagnosed, relapses or progression of the disease may occur. 2 ^,^ 32

IgA nephropathy (IgAN) is the most common form of primary glomerulonephritis in women in childbearing age.^35^^-^ 37 Proteinuria is determinant for pregnancy outcome in women with IgA nephropathy. The presence of >1g/day of proteinuria is associated with loss of permanent renal function. This feature is also associated with the decrease of birthweight.^33^^,^ 36 ^,^ 37

Other common forms of primary glomerulonephritis with less documented studies are focal segmental glomerulosclerosis, minimal change nephropathy, membranous glomerulonephropathy, and membrano-proliferative glomerulonephritis. 32 ^,^ 36

Although there is limited data on most primary glomerulonephritis, the available evidence agrees that the association of hypertension, proteinuria, and impairment of renal function are predictors of worse pregnancy outcomes.^7^^,^ 33 ^,^ 36 ^,^ 37 The use of acetylsalicylic acid earlier in pregnancy is recommended by some authors to improve placentation in primary and secondary glomerulonephritis.^35^^,^ 38

#### Systemic Lupus Erythematosus (SLE)

SLE is a chronic inflammatory autoimmune disease with a high prevalence in women of reproductive age, with lupus nephritis affecting approximately 50% of cases. This disease can arise for the first time in pregnancy. The physiological modifications of this period can influence the course of SLE and its renal manifestations.^14^^,^ 36 ^,^ 39 ^,^ 40

Nowadays, pregnancy with lupus nephritis is successful in many cases due to improved treatment strategies and to recognizing the importance of inducing and maintaining disease remission prior to conception - recommended for at least 6 months in order to reduce flares during pregnancy. A multidisciplinary team is vital for the successful management of lupus nephritis and pregnancy (including obstetricians, rheumatologists, and nephrologists).^36^^,^ 39 ^,^ 41

Absolute contraindications to pregnancy include severe pulmonary hypertension, restrictive lung disease, and heart failure. Severe CKD with serum creatinine > 2.5 mg/dL (CKD stages 3-5) represents a relative contraindication to pregnancy.^32^^,^ 39 ^,^ 42

The use of acetylsalicylic acid (100-150mg) during pregnancy is recommended in all women with lupus nephritis. Women with SLE and antiphospholipid syndrome should be additionally administered low molecular-weight heparin in prophylactic dosage to prevent adverse obstetrical and fetal outcomes.^36^^,^ 39 ^,^ 41

Neonatal lupus syndrome can occur with cutaneous, hematological, and cardiac manifestations (i.e. congenital heart block). This is an uncommon complication associated with the presence of maternal antibodies against intracellular ribonucleoproteins for Sjogren syndrome type A antigen (SSA) and Sjogren syndrome type B antigen (SSB) transported across the transplacentary barrier. Screening with fetal echocardiogram is recommended for patients positive for these antibodies and with suspected fetal dysrhythmia or myocarditis. The screening can begin at 16-18 weeks for high-risk patients (previous child with neonatal lupus or congenital heart block) and is recommended to be weekly from weeks 18-26 and every 2 weeks until week 32.^32^^,^ 41 ^-^ 43

The risk for complications (preeclampsia, preterm birth, low birthweight, fetal loss, flares) is present throughout all stages of renal disease. Fetal ultrasound surveillance is recommended with the first (11-14 weeks of gestation) and second trimester (20-24 weeks) ultrasounds, and after additional scans at approximately 4-week intervals until birth (or closer if suspicion of FGR or PE).^40^^,^ 41

The risk for flares of disease activity is increased in pregnancy and also in puerperium. For this reason, post-partum follow-up should be intensified in the first 6 to 12 months.^36^^,^ 41 ^,^ 43

#### Autosomal Dominant Polycystic Kidney Disease (ADPKD)

Autosomal dominant polycystic kidney disease is one of the most common genetic disorders in the world (mutation of PKD1 or PKD2 genes), with an estimated prevalence of 4 in 10,000.^44^^-^ 46

The diagnosis of this disease is more frequent after the third decade of life, since clinical manifestations are rare before this age, although these patients progressively develop renal cysts since early in life and can develop ESRD latter.^36^^,^ 44 ^,^ 47

Pregnant women with ADPKD have increased risk for pyelonephritis, PT birth, and PE, regardless of the presence of previous proteinuria or hypertension.^47^^,^ 48

Due to the autosomal dominant nature of the disease, there is a 50% risk of transmission to the offspring. Genetic counselling should be offered before conception and during pregnancy to all patients.^45^^,^ 49

Pre-implantation genetic diagnosis and prenatal fetal genetic diagnosis are currently available to avoid the transmission of ADPKD in cases with previously identified pathogenic gene mutation.^50^^,^ 51

Although studies previously advocated cesarean section delivery in all cases, more recently, some study groups limit this indication to women with large cysts and recent or massive bleeding.^14^^,^ 32

#### Diabetes Kidney Disease

Diabetes mellitus prevalence is progressively growing, and this disease is nowadays the main cause of ESRD worldwide.^52^ Patients with this disease can have micro and macrovascular complications, including diabetic nephropathy, affecting 5-10% pregnancies in women with type 1 diabetes mellitus.^36^

Diabetic nephropathy is associated with a 2 to 4-time increased risk of complications in pregnancy (PE, PT birth) mentioned before and, additionally, it is associated with a higher risk for congenital malformations (cardiac and neural tube defects) and perinatal death.^52^^,^ 53

Interventions such as renin-angiotensin-aldosterone system (RAAS) blockers or a protein-restrictive diet cannot be continued during pregnancy, but some studies suggest a reduction on adverse pregnancy outcomes and comorbidities with maintenance of this therapy until the pregnancy is confirmed.^54^

Counselling to achieve optimal glucose and hypertension control before pregnancy is fundamental to improve pregnancy outcomes and minimize the probability for congenital malformations, since a linear increase of these complications is described for higher levels of hemoglobin A1c.^55^

The aim is to achieve pre-conception glycemic control and maintaining it during pregnancy and post-partum, although this can be highly challenging due to the hormonal environment during pregnancy, which increases insulin resistance and consequently insulin dose requirement.^55^^,^ 56

### Comorbidities and Progression of Underlying Kidney Disease

#### Hypertension (HTA) And Hypertensive Disorders (Pe And Hellp)

Chronic hypertension is inarguably the most common comorbidity of CKD, affecting 20-50% of pregnant woman with this condition.^28^ The presence of hypertension in CKD increases substantially the risk for complications during pregnancy.

The risk of developing hypertension during pregnancy is more common in specific kidney diseases, such as diabetes nephropathy, ADPKD, and glomerulonephritis. This risk also increases throughout the stages of CKD, as well as the probability of deteriorating pre-existing HTA is higher.^57^

Regardless of being a new onset or chronic hypertension, this condition must be controlled and treated with appropriate medication (Table 2). Current literature supports a tight control of diastolic pressure (85 mmHg vs. 100 mmHg) in the general pregnant population with HTA, as this does not increase adverse pregnancy outcomes as previously believed.^58^ More recently, experts on CKD and pregnancy recommend this approach, although more studies need to be developed to reproduce this effect in this specific population.^7^^,^ 59

**Table 2:** Anti-hypertensive drugs used on CKD patients on conception, pregnancy and lactation

DRUGS	Conception	Pregnancy			Lactation
	Safe/Unsafe	Safe/Unsafe (FDA)	Maternal effects	Fetal effects	Safe/Unsafe
**ANTI-HIPERTENSIVE DRUGS**					
**FIRST CHOICE**					
Labetalol	Safe	Safe (C)	None	No teratogenicity	Safe
Nifedipine	Safe	Safe (C)	None	No teratogenicity	Safe
Alpha-methyldopa	Safe	Safe (B)	None	No teratogenicity	Avoid. Risk of post-partum depression
**SECOND-CHOICE DRUGS**					
Beta blockers	Safe	Safe (metoprolol –C Pindolol – B Atenolol -D)	May induce hypoglycaemia, hypotension and bradycardia (usually mild and transient) at delivery	FGR in some studies; Fetal bradycardia with atenolol in first trimester	Safe
Clonidine	Safe	Safe (C)	More common side effects and hypertensive rebound at discontinuation	FGR (uncommon)	Safe
Diuretics	Safe	Safe – use with caution (Hydrochlorothia-zide – B Amiloride - B)	Thiazides may be continued in patients previously on treatment. Amiloride – used in selected cases with Gitelman syndrome	Provokes diuresis in fetus	Safe
**TO BE AVOIDED**					
Angiotensin-converting enzyme inhibitors	Safe until 1^st^ trimester if required for nephroprotection	Unsafe (D)	None	Second and third trimester teratogenicity includes oligohydramnios, neonatal anuria and renal failure, limb contractures, craniofacial abnormalities, pulmonary hypoplasia, and patent ductus arteriosus	Safety data available for captopril and enalapril
Angiotensin receptor blockers	Unsafe (insufficient data)	Unsafe (D)			Insufficient data

Preeclampsia is a condition characterized by hypertension identified for the first time and significant end-organ dysfunction (thrombocytopenia, renal insufficiency, impaired liver function, pulmonary edema, new-onset headache not accounted for by alternative diagnoses) with or without proteinuria in the last half of pregnancy (>20 weeks of gestation) or postpartum. Preeclampsia is considered superimposed when it complicates pre-existing chronic hypertension. Women with kidney failure and earlier stages of CKD, including kidney transplant recipients, have a noteworthy higher risk for developing PE during pregnancy.^20^^,^ 60

HELLP syndrome is considered a severe form of PE, although some authors still defend it to be a separate disorder.^61^ The predominant features of this syndrome include hemolysis, elevated liver enzymes, and thrombocytopenia, rather than hypertension or central nervous system or renal dysfunction, although the latter also occurs. HELLP syndrome may have an insidious and atypical onset, with up to 15% of the patients lacking either hypertension or proteinuria.^60^^,^ 62

#### Proteinuria

Increased protein excretion can occur in healthy pregnancies due to the physiological changes that lead to an increase in glomerular filtration.^63^ In CKD, values of proteinuria above 3 g/dL are associated with acute and chronic glomerular disease, and prophylaxis with low-molecular-weight heparin in nephrotic patients (due to increased thrombotic risk in pregnancy) and acetylsalicylate acid in all patients with any degree of proteinuria is recommended.^32^^,^ 38

Proteinuria and/or hypertension lead to an increased risk for adverse fetal and maternal outcomes in pregnancy (including PE).^11^^,^ 64 Therapeutic agents that decrease the rate of protein excretion such as angiotensin-converting enzyme (ACE) inhibitors and angiotensin II receptor inhibitors have demonstrated teratogenic effects and cannot be used during pregnancy.^65^^,^ 66

The distinction between proteinuria of renal disease and proteinuria due to preeclampsia can be problematic due to the different management strategies associated to each pathology. Elements useful to this distinction are gestational age (PE is less probable before 20 weeks), quantification of protein excretion in early pregnancy in women at risk for kidney disease (chronic hypertension, diabetes mellitus, and systemic lupus erythematosus), and new angiogenic and antiangiogenic biomarkers (see Diagnostic challenges of CKD in pregnancy below).^67^^,^ 68

Low-protein diet can improve moderately the hyperfiltration effect of pregnancy and may be safely implemented in pregnant patients with CKD and proteinuria.^69^

#### Anemia

Anemia in CKD can have different causes such as disorders in iron homeostasis (via hepcidin excess) or deficiency of erythropoietin (EPO), which is one of the main regulators of red blood cells production.^70^^,^ 71 This molecule is originated in fibroblast-like cells in the kidney and owing to the increased needs of plasma cells in pregnancy its production is doubled. 

In CKD, the gestational increase in plasma volume is not accompanied by similar increase in red blood mass, and hemoglobin levels fall due to hemodilution.^70^^,^ 72 Anemia in CKD is associated with increased hospitalizations, cognitive impairment, and mortality, and in pregnancy, it is associated with adverse fetal outcomes such as prematurity and low birthweight.^70^^,^ 73

The treatment of anemia is essential for the prevention of maternal-fetal adverse outcomes. EPO is a large molecule and does not cross the placental barrier. Supplementation with synthetic EPO is relatively safe in pregnancy and may be required even in early stages of CKD (Table 1).^74^

Iron deficiency can also be involved in anemia in pregnancy and CKD, and iron supplementation may be necessary additionally to EPO.^75^ Current literature supports a more efficient therapeutic response with intravenous iron, especially in higher stages of the disease, but oral iron is a highly safe and efficient way of supplementation.^73^

#### Fetal Outcomes - Surveillance, Risks, and Delivery

Kidney disease can adversely affect pregnancy even in early stages.^28^ Neonates of mothers with CKD, compared to normal mothers, are at risk for preterm birth (20% to 50%), FGR (five times higher), small for gestational age infants (three times higher), neonatal mortality (five times higher), stillbirths (9 times higher), and low birth weight (fivefold higher).^26^^,^ 76 ^,^ 77

Surveillance should include first and second trimester screening, followed by bi-weekly growth scans after 28-30 weeks combined with Doppler studies to detect FGR in an early stage.^13^^,^ 78 ^,^ 79

Serum human chorionic gonadotropin is both part of first and second trimester screening. In advanced CKD there can be higher serum levels of this hormone due to its deficient excretion by the kidney. Consequently, false-positive tests raise the need for other pre-natal diagnosis alternatives (cell-free DNA, chorionic villous sampling, or amniocentesis).^80^^,^ 81

Cardiotocography is an important instrument in fetal evaluation and should be considered in the surveillance of pregnancy with CKD.^13^^,^ 78

Delivery should be individualized according to the different complications of pregnancy. Similarly to normal pregnancies, elective delivery is indicated if labor has not occurred by the estimated date for delivery (39 to 40 weeks).^82^^,^ 83 If a hypertensive disorder is present, recent studies support expectant management for women with non-severe hypertension until 37 weeks of gestation. Indications for termination of pregnancy include uncontrollable hypertension with or without superimposed PE, severe FGR, and modifications in fetal biophysical profile.^13^^,^ 78 ^,^ 84 ^,^ 85

If significant/progressive aggravation of maternal renal function is verified, an individualized decision between termination of pregnancy (in early stages), delivery, and initiating dialysis (in specific circumstances) must be discussed between patient and the medical team.^63^^,^ 86

Concerning delivery, CKD is not a contraindication to vaginal delivery, and this is the preferred method of delivery if no other indication for cesarean section is present.^86^^,^ 87

#### Diagnostic Challenges of CKD in Pregnancy (Biomarkers, Ultrasound, Kidney Biopsy)

The differential diagnosis between CKD and preeclampsia remains challenging due to the overlap of symptoms (hypertension) and analytic parameters (significant proteinuria). Efforts have been made to study possible new biomarkers and ultrasound parameters to help in the diagnosis of these pathologies.^57^^,^ 68

Regarding ultrasound studies, recent literature shows that abnormal flow of the uterine (altered resistance index or early diastolic notching) and umbilical arteries (altered pulsatility index or abnormal patterns of umbilical artery Doppler waveforms, especially absence or reversal of end-diastolic velocities) are highly suggestive of preeclampsia and, on the other end, normal flow of both vessels are more suggestive of CKD, in the presence of hypertension and proteinuria. These findings need further confirmation.^68^

The ratio between antiangiogenic biomarkers such as soluble FMS-like tyrosine kinase-1 (sFLT1) and angiogenic markers such as placental growth factor (PLGF) was demonstrated to usefully predict preeclampsia in gestations complicated by hypertension and the need for more or less urgent action.^27^^,^ 67 ^,^ 88 ^,^ 89

Regarding CKD, these biomarkers follow the same gestational pattern as in women without pre-existing disease, which supports the substantial contribution of placental insufficiency in the pathogenesis of PE, and consequently this ratio can be a helpful tool in the distinction between CKD and this hypertensive disorder.^27^^,^ 89

Kidney biopsy is not contraindicated during pregnancy and can be a helpful tool in defining the course of treatment and pregnancy surveillance, although a higher rate of complications of this procedure can occur especially around 25 weeks of gestation.^90^^-^ 92 Due to the advanced uterine growth and limited benefits of diagnosis in late pregnancy, it is advisable to avoid biopsies after 30 weeks of gestation.

The decision for this procedure should be individualized and it is recommended to do the biopsy preconceptionally or in early pregnancy (before 25 weeks) to reduce its complications.^63^^,^ 93 ^,^ 94

#### Therapeutic Management - Drugs, Dialysis and Transplantation

The management of drugs in pregnancy with CKD presents a challenge. In order to reduce pregnancy complications in kidney disease, independently of comorbidities, therapeutic modifications must start at preconception (Table 1 and 2).^95^^,^ 96

Several medications used to control hypertension, in the management of autoimmune diseases, or for the prevention of transplant rejection in CKD patients can have teratogenic effects and must be discontinued or changed before or at the beginning of pregnancy.^6^^,^ 95

In addition, prenatal supplementation is essential and must be initiated before conception in adequate doses (4 mg/day folic acid and 200 mcg/day iodine in women without thyroid disease).^97^^,^ 98

#### Antihypertensive Drugs

Achieving blood pressure control before and during pregnancy is a priority to avoid complications such as preeclampsia. Different classes of medications can be used, but older classes of antihypertensive drugs such as labetalol, nifedipine, metildopa are considered safer during pregnancy, and are first line drugs used in the control of blood pressure during this period.^57^^,^ 63 ^,^ 96

Drugs that can block the RAAS such as ACE inhibitors and angiotensin receptor blockers are able to provide nephroprotection and delay the progression of renal disease. These medications can pass the placental barrier and are fetotoxic mostly in the second and third trimester, causing intrauterine growth restriction, renal dysplasia, oligohydramnios, and fetal death. 13 ^,^ 29 ^,^ 99

In compliant women with regular menstrual cycles, there is the possibility to continue this medication until a positive pregnancy test or during the first weeks of pregnancy to offer additional nephroprotection.^14^^,^ 100

#### Immunosuppressive Drugs

An autoimmune component is behind several kidney diseases that can lead to CKD (SLE, primary glomerulonephritis), and immunosuppressive drugs can help induct or maintain the control of these pathologies. This class of medication is used also in transplant patients to prevent graft rejection.^92^

Medications such as steroids, azathioprine, and calcineurin inhibitors (cyclosporine and tacrolimus) have a good safety profile and can be used in pregnancy.^29^^,^ 31

Prednisone is recommended, as only a small fraction of this steroid passes the placental barrier. Women taking doses superior to 5 mg per day for more than 3 weeks in pregnancy in the six months prior to delivery may have suppression of hypothalamic-pituitary-adrenal function, and administration of intravenous steroids at the time of delivery to cover the stress caused by this process can be considered.^14^^,^ 87 ^,^ 92

Some immunosuppressants are known for its teratogenic effects and should be discontinued or shifted, ideally 3 months before pregnancy, in order to adjust doses of the new medication and evaluate maintenance of remission.^19^^,^ 29 ^,^ 92

Mycophenolate mofetil and cyclophosphamide are teratogenic during pregnancy.^31^^,^ 101 ^,^ 102 Mycophenolate mofetil can be used in patients with LES to prevent flares and also after renal transplantation, but it has a high risk of miscarriage and a known pattern of fetal toxicity that can cause hypoplastic nails, shortened fingers, micrognathia, cleft lip and palate, diaphragmatic hernia, and congenital heart defects.^101^^,^ 103 Cyclophosphamide is an alkylating agent used in patients with rapidly progressive glomerulonephritis due to potent immunosuppressant effect. The use of this drug is contraindicated in pregnancy and lactation, as it is transferred through the placenta and to maternal milk, causing congenital abnormalities of the skull, ear, face, limbs, and visceral organs of the fetus.^102^^,^ 104

#### Biologic Agents

Autoimmune and/or inflammatory diseases can have renal manifestations that lead to CKD (e.g. SLE and glomerulopathies). Studies of the different inflammatory pathways and mediators that cause these diseases have led to critical advances in the treatment of these conditions. Several new biologic agents have been introduced into the therapy of autoimmune diseases and improved the outcome of patients (e.g. rituximab in membranous glomerulonephritis and SLE, belimumab in SLE).^105^^-^ 107

Biologic agents are derivatives of IgG, differing in structure, half-life, and placental passage. Placental transfer of IgG is limited during organogenesis, but increases gradually and exponentially from the beginning of the second trimester until term (Table 1).^105^^,^ 108

Limited information is available regarding the safety of these agents in pregnancy. The different agents have distinct recommendations and studies regarding use during preconception, pregnancy, and lactation. Decisions should be individualized considering the possible risks of these medications (i.e. risk of opportunistic infection, structural malformations, miscarriage, premature birth) vs. the risk of maternal disease relapse due to discontinuation of these therapeutic agents.^31^^,^ 105

#### Other Medications Used in Pregnancy

Hydroxychloroquine is an immunomodulator drug used in SLE to prevent flares with known safety data during pregnancy and lactation. It crosses the placental barrier but is not associated with fetal toxicity. It is also associated with evidence of improving placentation and preventing FGR and heart block.^29^^,^ 41

Aspirin use during pregnancy in CKD is highly recommended from 12 weeks of gestation to 36 weeks. This drug was demonstrated to reduce preeclampsia rates during pregnancy, with no additional hemorrhagic side effects.^29^^,^ 57 ^,^ 96

In patients with increased proteinuria (threshold still undefined), there is a higher risk for thrombotic events. In pregnant women with proteinuria, low-molecular weight heparin is recommended and has a safe profile since there is little to none placental transfer.^13^^,^ 29 ^,^ 96

#### Dialysis

In the past, pregnancy was contraindicated in women undergoing renal replacement therapy. Presently, we observe an increase of pregnancies of women under dialysis due to many factors such as increase of maternal age, advances in the delivery of chronic dialysis, and limited availability of organs for transplant.^109^^-^ 111

The direct relation between glomerular filtration rate and fertility and sexual dysfunction is well established.^19^^,^ 22 Studies have shown that the percentage of women under dialysis with regular menstruation cycles (42%) is inferior when compared with women with CKD prior to dialysis (75%). A significant percentage of women under dialysis is amenorrheic (37-60%).^23^

However, this fertility impairment does not eliminate the need for efficient contraception.^109^ Emerging evidence is showing that increasing the hours of dialysis (from 16.5 hours to 28 hours) can lead to the return of menstrual cycles in previously amenorrheic women, and further intensification of the provision of dialysis can increase conception rates up to 15.6%.^109^^,^ 112

In pregnant women with CKD, the indications to start dialysis during pregnancy are mostly the same as in other patients:

1. Metabolic and electrolyte changes that cannot be resolved with drug therapy;
2. Pregnant women with residual renal function and creatinine clearance <20 mL/min/1.73m^2^, with confirmed progressive loss of kidney function, or women in which urea levels consistently exceed 50-60 mg/dL (18-21 mmol/L).

These patients should be considered for hemodialysis, due to the adverse fetal outcomes associated with increased urea concentrations, which can further compromise the pregnancy.^86^^,^ 109 ^,^ 110 ^,^ 113 ^,^ 114 This differs from the approach in non-pregnant CKD patients in whom there is no minimum GFR or threshold urea levels that provide an absolute indication to begin dialysis in the absence of symptoms. However, this decision must always be individualized.^113^^,^ 114

The rate of live-births under dialysis improved in the last decades from 25% in 1960 to >75% in the present years, although 53.4% of babies were born preterm and 65% had low birthweight (<2.5 kg).^115^ This is owed to the provision of intensified dialysis with daily and overnight regimens of hemodialysis (HD) and intensified peritoneal dialysis (PD), aiming for at least 36 hours per week (5-6 sessions/week) for women without residual renal clearance.^109^^,^ 110 ^,^ 114 ^,^ 116 Several recent studies support this finding and show that increases in the number of hours of hemodialysis are inversely related to the rate of preterm birth due to improved volume management and clearance of blood urea nitrogen and other solutes. Levels of uremic toxins are directly correlated to fetal mortality, with no documented live births with urea levels >60 mg/dL (21.4 mmol/L). Treatment targets for urea are set in near-normal urea levels of approximately 28-42 mg/dL (10-15 mmol/L) predialysis.^86^^,^ 109 ^,^ 110 ^,^ 114 ^,^ 116

Additionally, intensified dialysis allows an improved control of interdialytic weight gain and better blood pressure control with fewer hypotensive episodes. The reduction of maternal hemodynamic instability with this dialytic regimen is fundamental to avoid compromise to the uterus-placental circulation.^86^ The rate of PE in pregnant women under dialysis was 19.4%. 86 ^,^ 115

PD during pregnancy may require adjustments in order to prevent volume overload and high vigilance for signs of peritonitis. A considerable percentage of women switch from PD to HD during pregnancy since data is limited on the advantages of PD throughout this period.^13^^,^ 117

The rate of complications is still high despite the start of dialysis early in pregnancy, although it is not incompatible with successful pregnancy outcomes.^88^^,^ 110

#### Transplantation

Pregnancy in renal transplant recipients is relatively uncommon (5 cases in 100,000 births).^118^ Transplantation increases the possibility of a live birth by 10-fold in pregnant women with CKD compared with dialysis.^119^^,^ 120

**Figure 1:**
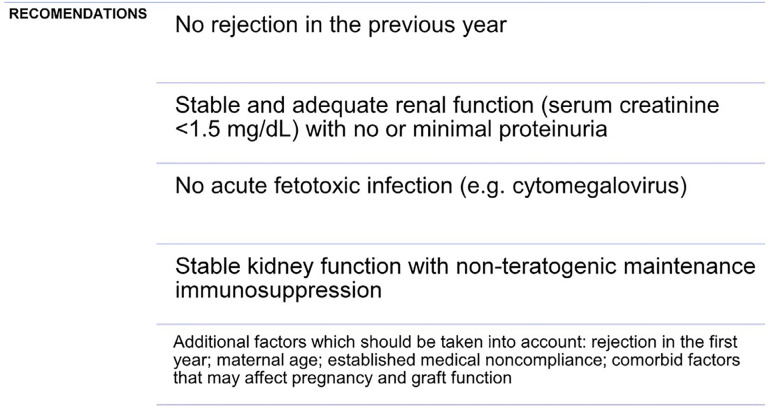
Recommendations of the American Society of Transplantations to be followed before conception.

There is no predefined timing for conception and evidence is limited, but most studies report that women are advised to wait at least 1 year, and a few criteria must be ensured before pregnancy^121^^,^ 122 (Figure 1).

Although the live birth rate is superior in women with renal transplant (75-80%) compared to dialysis, these pregnancies still have a higher rate of complications despite reinstatement of renal function.^89^^,^ 91 The most common complications are hypertension, preeclampsia (26%), prematurity (46%), small for gestational age newborn (54%), higher rates of cesarean section (53-72%), and loss of graft function (27-34%).^14^^,^ 121 ^,^ 123

Risk factors for poor maternal and fetal outcomes include absence of pre-pregnancy stable renal function with elevated serum creatinine (>1.5 mg/dL), and it is demonstrated in current literature that higher rates of loss of graft function occur in these women. Counselling of women concerning graft longevity is indispensable when considering a pregnancy.^120^^,^ 124

#### Follow-Up of Pregnancy in CKD Patients

**Figure 2:**
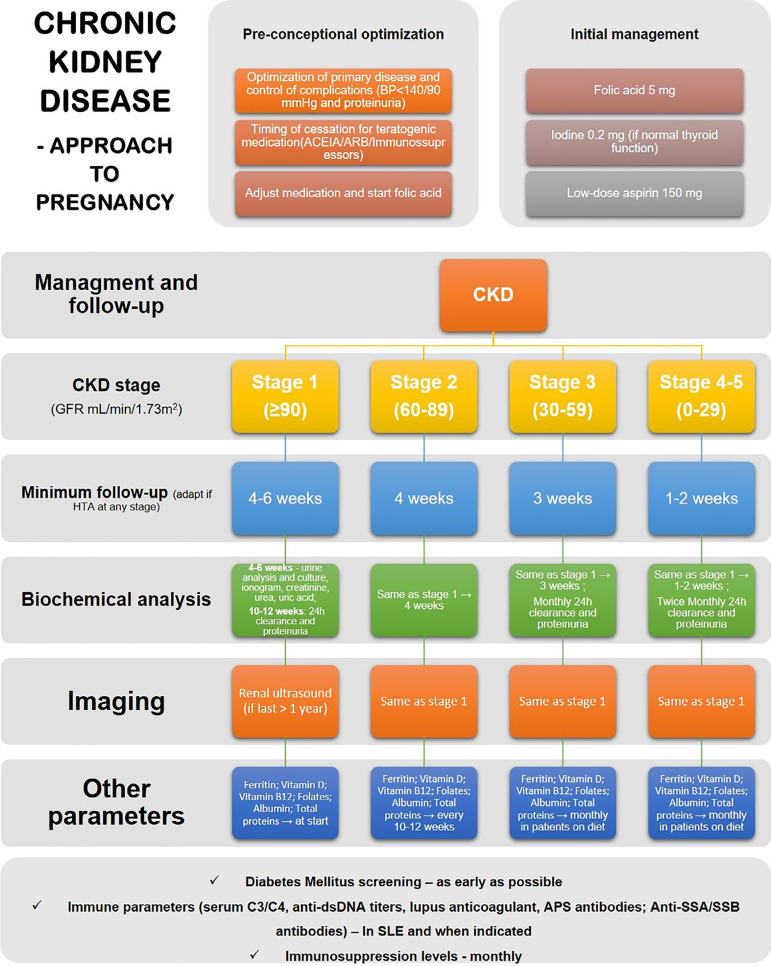
Chronic kidney disease in pregnancy - suggested management based on the Italian group approach ACEI, angiotensin-converting enzyme inhibitor; ARB, angiotensin receptor blocker; CKD, chronic kidney disease; SLE Systemic lupus erythematosus.

Pregnancy in CKD patients requires a systematic follow-up regimen in order to promptly adapt CKD therapies to this state and identify any maternal or fetal complications before or early on the gestation. Current literature supports intensification of follow-up with the increase in CKD stage and also the appearance of comorbidities such as hypertension, proteinuria, and systemic disease (Figure 2).^32^^,^ 63 ^,^ 87

## Conclusions

Pregnancy in women with CKD is becoming more frequent and requires exhaustive counselling and planning from preconception to delivery. In order to accomplish a careful follow-up and achieve successful outcomes, it is vital to reunite a multidisciplinary team. Women should be advised of the risks of pregnancy in CKD, and therapeutic and emotional support must be provided throughout the different stages of gestation.

Although the rate of successful pregnancies in CKD has improved through the years, it is essential to take into account the high number of pregnancies complicated by preterm birth, hypertension, preeclampsia, and FGR, even after transplantation and apply the correct measures do decrease these hazards. Education of patients by the medical team (obstetrics and nephrologists) is mandatory to avoid unplanned pregnancies and achieve conception during a certain window of opportunity, thus improving maternal and fetal outcomes.
